# Association of Ankle–Brachial Index with Quality of Life and Survival Outcomes in Hemodialysis Patients

**DOI:** 10.3390/jcm14051625

**Published:** 2025-02-27

**Authors:** Norihito Yoshida, Tatsuki Tanaka, Yusuke Suzuki, Sadamu Takahashi, Mai Hitaka, Shingo Ishii, Keisuke Yamazaki, Yasushi Ohashi

**Affiliations:** Department of Nephrology, Toho University Sakura Medical Center, Chiba 285-8741, Japan; norihito.yoshida@med.toho-u.ac.jp (N.Y.); tatsuki.tanaka@med.toho-u.ac.jp (T.T.); yusuke.suzuki@med.toho-u.ac.jp (Y.S.); sadamu.takahashi@med.toho-u.ac.jp (S.T.); mai.hitaka@med.toho-u.ac.jp (M.H.); shingo.ishii@med.toho-u.ac.jp (S.I.); keisuke.yamazaki@med.toho-u.ac.jp (K.Y.)

**Keywords:** peripheral arterial disease, ankle–brachial index, quality of life, hemodialysis, malnutrition, body composition analysis

## Abstract

**Background/Objectives**: Ankle–brachial index (ABI) is frequently measured in hemodialysis patients due to their high cardiovascular risk, while its potential role as a screening tool for assessing overall physical function and health-related quality of life (QOL) remains unclear. This study aimed to evaluate the association of the ABI with QOL and survival in hemodialysis patients. **Methods**: This study included 346 hemodialysis patients, categorized into two groups based on their ABI (≤0.9 vs. >0.9). Clinical parameters, QOL (measured using SF-36 and KDQOL questionnaires), and survival outcomes were analyzed. **Results**: There were 66 (19.1%) patients with an ABI ≤ 0.9 in this study population. Patients with an ABI ≤ 0.9 exhibited significantly older ages, longer dialysis durations, higher prevalence of diabetes mellites and cardiovascular disease, elevated N-terminal pro-brain natriuretic peptide levels, and higher calcitriol use but lower phase angle, skeletal muscle mass index values, health-related QOL domains, and several kidney disease-specific QOL domains compared to those with an ABI > 0.9. Kaplan–Meier analysis revealed significantly higher cumulative mortality in the ABI ≤ 0.9 group (6.6 vs. 2.5 per 100 patient-years, *p* < 0.001). **Conclusions**: A low ABI is significantly associated with decreased QOL and higher mortality risk in hemodialysis patients. While traditionally used for PAD screening, the ABI may serve as a practical tool for predicting QOL decline and survival outcomes. Interestingly, the ABI was also linked to muscle attenuation and volume overload. ABI assessment could aid in early risk stratification and guide multidisciplinary interventions, including exercise programs, nutritional support, and cardiovascular risk management, to improve patient care and outcomes.

## 1. Introduction

As of the end of 2020, there were approximately 340,000 hemodialysis patients in Japan, with an average age of 69.4 years. The number of dialysis patients over 70 years old is steadily increasing [1]. Hemodialysis requires patients to undergo center treatment three times a week, with each session lasting about four hours. Despite these time constraints, dialysis is not a perfect substitute for kidney function, and patients must also adhere to strict dietary and fluid intake restrictions. Many dialysis patients suffer from multiple comorbidities and complications related to dialysis, and their perceived health status is significantly lower compared to the general population [2]. Improving the quality of life (QOL) for dialysis patients, despite the daily limitations imposed by their treatment, remains a significant clinical challenge.

Cardiovascular disease is the most critical prognostic complication among dialysis patients, and peripheral arterial disease (PAD) is also prevalent. Patients with PAD are generally known to have both a poorer prognosis and a diminished QOL [3,4,5]. PAD typically follows a chronic course, leading to lower extremity pain and impaired mobility. The progression of PAD is primarily attributed to factors such as hypertension, diabetes mellitus (DM), chronic inflammatory states leading to malnutrition, vascular calcification associated with aging and abnormal calcium–phosphorus metabolism, and the accumulation of uremic toxins [6]. Hemodialysis patients often present with multiple risk factors for PAD [7]. The ankle–brachial index (ABI) is a widely used, non-invasive, and relatively simple test for the primary screening of PAD [8]. The ABI has been reported to correlate with cardiovascular events and mortality [9,10,11]. A recent study has shown that in the general population, a lower ABI is significantly associated with poorer QOL scores, regardless of the presence or absence of intermittent claudication [12]. This suggests that the ABI may reflect systemic vascular dysfunction and physical deterioration beyond its traditional role in PAD detection. Otherwise, the adverse outcomes in patients with a low ABI may be multifactorial [13]. Despite extensive research on the ABI in cardiovascular risk assessment, limited evidence exists regarding its utility in predicting QOL deterioration in hemodialysis patients [12]. Given that the ABI is frequently measured in this population due to their high cardiovascular risk, its potential role as a screening tool for assessing overall physical function and health-related QOL warrants further investigation. Therefore, this study aims to clarify the relationship between the ABI and QOL in patients undergoing chronic maintenance hemodialysis and to assess whether the ABI can be utilized as a simple and effective tool for predicting QOL decline and survival outcomes. By leveraging the ABI as an early indicator of declining QOL and increased mortality risk, targeted interventions may be implemented to improve daily living and treatment satisfaction in this population.

## 2. Materials and Methods

### 2.1. Study Design and Participants

**Figure 1:**
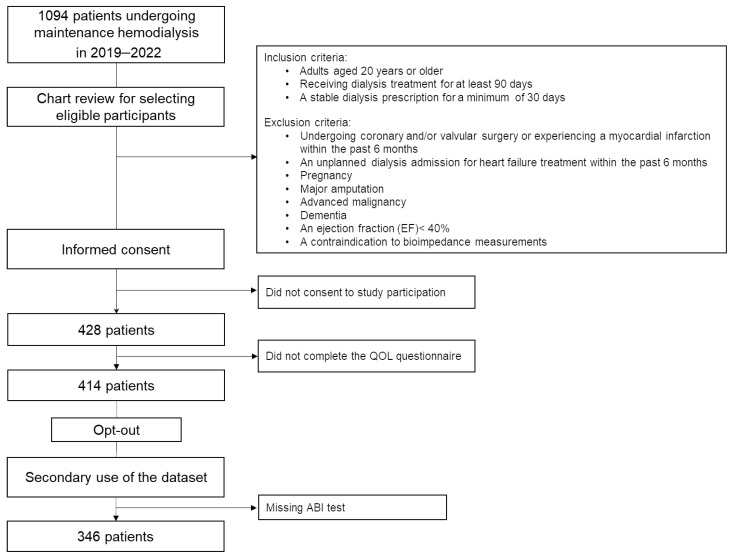
Flow chart of the study participation process.

Between 2019 and 2022, a multicenter cross-sectional study was conducted across four chronic hemodialysis clinics: Seijinkai Mihama Hospital, Seijinkai Mihama Sakura Clinic, Seijinkai Narita Clinic, and Seijinkai Katori Clinic (approved by the ethics committee of Toho University Sakura Medical Center, Tokyo, Japan: approval no. S21073|26 April 2022 [S18086|27 December 2018]). As shown in Figure 1, 1094 patients were undergoing hemodialysis at these facilities (mean age: 68 ± 13 years; 777 men and 317 women). Eligible participants were selected from patients receiving daytime dialysis, and 428 patients were identified from electronic medical records. Inclusion criteria consisted of adults aged 20 years or older who had been receiving dialysis treatment for at least 90 days and who had maintained a stable dialysis prescription for a minimum of 30 days at the time of recruitment. Exclusion criteria included the following: undergoing coronary and/or valvular surgery or experiencing a myocardial infarction within the past 6 months; an unplanned dialysis admission for heart failure treatment within the past 6 months; pregnancy; major amputation, advanced malignancy, or dementia; echocardiography within the past year showing an ejection fraction (EF) < 40%; or missing data. Participants who consented to provide medical information and complete the QOL questionnaire were included in the study. For the present study, 346 patients who had undergone ABI testing during the same period were selected from this dataset. Research consent was obtained using an opt-out method, ensuring no objections, and this study was conducted as a retrospective cohort and cross-sectional study. Secondary use of the data was approved by the ethics committee of Toho University Sakura Medical Center (S24035).

### 2.2. Quality of Life Questionnaire

Participants completed the Kidney Disease Quality of Life-Short Form (KDQOL-SF) ver.1.3 questionnaire within the first 60 min of their dialysis session [14]. This revised version of the SF-36 is specifically designed for patients undergoing dialysis and has been validated for use with Japanese patients [15]. It includes renal disease-specific items that measure both overall quality of life and the quality of life specific to renal disease patients. The questionnaire comprises ten domains: symptoms, impact on daily life, burden of renal disease, work status, cognitive function, quality of social interaction, sleep, social support, encouragement from dialysis staff, and patient satisfaction. Additionally, the SF-36 health survey components assess physical functioning, role limitations due to physical health, bodily pain, general health, vitality, social functioning, role limitations due to emotional health, and mental health. The questionnaires were self-administered and distributed during dialysis sessions, with participants either returning to them at the next session or completing them during the same session. Nurses and clinical engineering technicians, who had been briefed on the questionnaire in advance, provided the forms along with notes and the study consent form. They also explained the questionnaire and its completion to participants who agreed to take part in the study.

### 2.3. Data Collection

Baseline clinical data were collected, including age, gender, the presence of diabetes, the duration of dialysis, comorbidities, body weight, pre-dialysis blood pressure, the history of phosphate binder use, and the history of active vitamin D supplementation. Blood tests were conducted at the beginning of each month for hemodialysis management, with the following standard laboratory parameters collected: blood urea nitrogen (BUN), serum creatinine, calcium, phosphorus, C-reactive protein, hemoglobin, and intact parathyroid hormone (iPTH) levels. Dialysis adequacy was assessed using the urea reduction ratio and single-pool Kt/Vurea, calculated using the Shinzato formula [16]. The Geriatric Nutritional Risk Index (GNRI) was calculated as (14.89 + albumin g/dL) + (41.7 × body weight/ideal body weight) [17]. Pre-dialysis N-terminal pro-brain natriuretic peptide (NT-proBNP) levels were measured using an electrochemiluminescence immunoassay system (Cobas 8000 e801 module; Roche Diagnostics K.K., Tokyo, Japan). Standard multi-frequency-bioimpedance analysis (MF-BIA) was performed with the patient in the supine position placed on a bed after hemodialysis. For body composition measurements, a segmental MF-BIA device (Inbody S10^®^; InBody Co., Ltd., Seoul, Republic of Korea) was used. Microprocessor-controlled switches and an impedance analyzer were activated, and segmental resistances of the arms, trunk, and legs were measured at four frequencies (5, 50, 250, and 500 kHz). Subsequently, total body water, intracellular water (ICW), extracellular water (ECW), and the ECW to ICW ratio were calculated using the InBody S10^®^ software. The phase angle was calculated based on the resistance (R) and reactance (Xc; measured at 50 kHz) using the following equation: PhA (°) = arctangent (Xc/R) × (180/π). The skeletal muscle mass index (SMI) (appendicular skeletal muscle mass/height^2^, kg/m^2^) was measured as the sum of the lean soft tissue of the two upper and lower limbs.

### 2.4. ABI Measurement Method

ABI values were collected from patients’ medical records, with the most recent measurement included in this study. The ABI was measured annually during the patient’s birth month, ensuring consistency in data collection. No cases of abnormally high ABI values (ABI > 1.4) were observed in this study population. The ABI was measured using the VS-2500 blood pressure pulse wave device (Fukuda Denshi Co., Ltd., Tokyo, Japan; certification number: 301ADBZX00035000), a validated tool commonly used for PAD screening. Measurements were performed in a supine position after a 5 min rest, with cuffs placed on both arms and ankles. To ensure consistency, ABI values were obtained from both sides, and the lower of the two ABI values was recorded for analysis to reflect the more severe vascular impairment. Patients were categorized into two groups using an ABI cutoff value of 0.9, based on established studies on PAD diagnostic sensitivity and cardiovascular event prediction. To minimize variability, measurements were taken from the non-shunt or non-superficialized limb whenever possible. Patients were categorized into two groups using an ABI cutoff value of 0.9, based on studies regarding the diagnostic sensitivity of PAD and the incidence of cardiovascular events [18,19,20,21]. Upper extremity measurements were conducted on the non-shunt limb or the non-superficialized limb.

### 2.5. Statistical Analysis

Data were analyzed using JMP Pro (version 16.0; SAS Institute Inc., Cary, NC, USA). The normality of ABI values was assessed using the Shapiro–Wilk test, which indicated that the data were not normally distributed (W = 0.934, *p* < 0.001). Therefore, nonparametric tests were applied to ensure robust statistical analysis. Clinical data were presented as medians with interquartile ranges (IQRs). Depending on the variable type, either the Wilcoxon rank-sum test (for continuous variables) or Pearson’s chi-square test (for categorical variables) were used for statistical comparison. Comparisons between the two groups were expressed as a mean difference or an odds ratio (OR). QOL scores were also reported as medians with IQRs and a 95% confidence interval (CI) in tables and as averages in figures. In this study, patients were categorized into two groups (ABI ≤ 0.9 and ABI > 0.9) based on their ABI values to examine its association with QOL and clinical outcomes. Bivariate analysis was first performed to evaluate the relationship between various factors and an ABI ≤ 0.9 in dialysis patients. The analysis identified that ABI values had a significant impact on QOL scores. Kaplan–Meier survival curves were generated to compare cumulative mortality between groups (ABI ≤ 0.9 vs. ABI > 0.9), and statistical significance was determined using the log-rank test. A *p*-value of less than 0.05 was considered statistically significant.

## 3. Results

### 3.1. Clinical Parameters

The median [(IQR), 95% CI] of the ABI was 1.05 (0.94–1.149, 1.00–1.04), with minimum and maximum values of 0.39 and 1.33, respectively. There was no significant difference in the ABI between right limbs and left limbs (1.06 vs. 1.06, *p* = 0.41). Patients with a bilateral ABI ≤ 0.9 and those with a unilateral ABI ≤ 0.9 numbered 30 and 36, respectively. The bilateral low ABI group exhibited more severe ABI values compared to the unilateral low ABI group (0.69 vs. 0.84, *p* < 0.001). Eventually, there were 66 (19.1%) patients with an ABI ≤ 0.9 in this study population. The comparison between patients with an ABI > 0.9 and an ABI ≤ 0.9 revealed significant differences in several clinical parameters (Table 1 and Table 2). Patients with an ABI ≤ 0.9 had a significantly higher median age of 70 years versus 66 years (mean difference: 4.8 years, 95% CI: 1.5–8.0, *p* < 0.001). The prevalence of DM was higher in the ABI ≤ 0.9 group compared to the ABI > 0.9 group (OR: 2.33, 95% CI: 1.33–4.00, *p* = 0.002), as was the prevalence of cardiovascular disease (OR: 2.44, 95% CI: 1.33–4.48, *p* = 0.005). Additionally, patients with an ABI ≤ 0.9 had a significantly longer hemodialysis duration, with a median of 100 months compared to 62 months, a mean difference of 55 months (95% CI: 31.26–79.30, *p* < 0.001). BUN levels were significantly lower in patients with an ABI ≤ 0.9, with a median of 54 mg/dL compared to 58 mg/dL, and HbA1c levels were significantly lower in the ABI ≤ 0.9 group, with a median of 5.9% compared to 6.3%. In contrast, NT-proBNP levels were significantly higher in patients with an ABI ≤ 0.9, with a median of 6050 pg/mL compared to 2985 pg/mL (mean difference: 5620 pg/mL, 95% CI: 2589–8650, *p* < 0.001). Interestingly, the number of phosphate binders was lower in patients with an ABI ≤ 0.9, but the proportion of calcitriol use was significantly higher in them compared to those with an ABI > 0.9 (*p* < 0.001).

### 3.2. Association of ABI ≤ 0.9 with Body Fluid Composition

The analysis of body fluid composition between dialysis patients with an ABI ≤ 0.9 and those with an ABI > 0.9, as presented in Table 2, revealed several significant differences. Patients with an ABI ≤ 0.9 had a lower amount of intracellular water per body surface area, with a median value of 11.8 L, compared to 12.2 L in the ABI > 0.9 group (mean difference −0.52, 95% CI: −0.90–−0.15, *p* = 0.006). Additionally, the extracellular-to-intracellular-water ratio was notably higher in the ABI ≤ 0.9 group, with a median ratio of 0.66 versus 0.63 in the ABI > 0.9 group (mean difference 0.02, 95% CI: 0.01–0.03, *p* < 0.001). Furthermore, the SMI was significantly reduced in patients with an ABI ≤ 0.9, with a median of 8.4 kg/m^2^ compared to 9.0 kg/m^2^ in the ABI > 0.9 group (mean difference −0.38, 95% CI: −0.76–−0.01, *p* = 0.007). This reduction in muscle mass indicates potential sarcopenia and muscle atrophy in the ABI ≤ 0.9 group. The phase angle, a marker of cell membrane integrity and cellular function, was also significantly lower in patients with an ABI ≤ 0.9, with a median of 4.8° versus 5.3° in the ABI > 0.9 group (mean difference −0.7, 95% CI −0.4–0.2, *p* < 0.001).

### 3.3. Association of ABI ≤ 0.9 with QOL Domains

**Figure 2:**
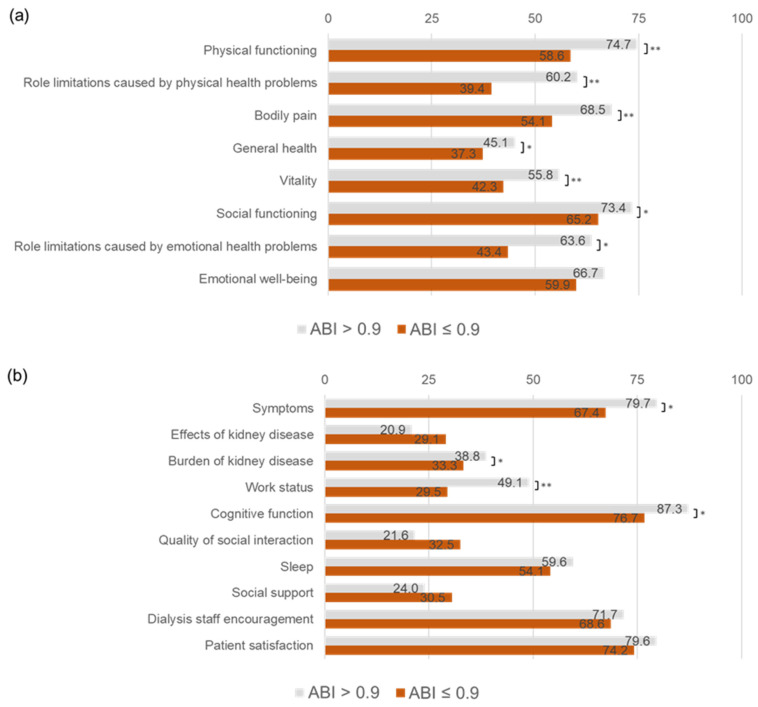
QOL domains with an ankle–brachial index ≤ 0.9 and with an ankle–brachial index > 0.9. (**a**) Health-related QOL domains: This panel compares scores for physical functioning, role limitations caused by physical and emotional health problems, bodily pain, general health, vitality, social functioning, and emotional well-being between patients with an ABI ≤ 0.9 and those with an ABI > 0.9. Patients with an ABI ≤ 0.9 had significantly lower scores in physical functioning, role limitations caused by physical health problems, bodily pain, general health, and vitality (** *p* < 0.001 or * *p* < 0.05), indicating a poorer health-related QOL. Refer to the Abbreviations section for definitions of all abbreviations used in the Figure. (**b**) Kidney disease-specific QOL domains: This panel compares scores for symptoms, effects and burden of kidney disease, work status, cognitive function, quality of social interaction, sleep, social support, dialysis staff encouragement, and patient satisfaction. Patients with an ABI ≤ 0.9 reported significantly lower scores in the burden of kidney disease, work status, and cognitive function (** *p* < 0.001 or * *p* < 0.05), highlighting the negative impact of a lower ABI on kidney disease-specific QOL. Refer to the Abbreviations section for definitions of all abbreviations used in the Figure.

The study compared various quality of life (QOL) domains between dialysis patients with an ABI > 0.9 and an ABI ≤ 0.9 (Table 3, Figure 2). Patients with an ABI ≤ 0.9 exhibited significantly lower scores across multiple domains, particularly in physical, mental, and kidney disease-specific QOL measures. Physical health domains were markedly impaired in patients with an ABI ≤ 0.9. Physical function scores were significantly lower, with a median of 65 compared to 80 (*p* < 0.001). Role limitations due to physical health were also more pronounced, with a median of 0 compared to 75 (*p* < 0.001). Additionally, bodily pain scores were lower (*p* < 0.001), and general health perception was worse, with a median of 40 vs. 50 (*p* = 0.004). Mental health and social domains were also negatively impacted in patients with an ABI ≤ 0.9. Vitality scores were significantly lower, with a median of 45 compared to 55 (*p* < 0.001). Social functioning scores were reduced, with a median of 63 vs. 75 (*p* = 0.048), as were role limitations due to emotional problems, with a median of 0 vs. 100 (*p* = 0.002). Kidney disease-specific QOL was significantly lower in several domains among patients with an ABI ≤ 0.9. Symptom burden scores were lower, with a median of 79 compared to 83 (*p* = 0.003). The burden of kidney disease was perceived to be greater, with a median of 31 vs. 38 (*p* = 0.039). Work-related QOL was significantly impaired, with a median of 25 vs. 50 (*p* < 0.001), along with cognitive function (median: 87 vs. 93, *p* = 0.017). The quality of social interaction, with a median of 87 vs. 93 (*p* = 0.24) and sleep scores, with median of 58 vs. 63 (*p* = 0.25), were lower in the ABI ≤ 0.9 group, although the differences were not statistically significant. Overall, these findings indicate that patients with an ABI ≤ 0.9 experience substantial impairments across multiple QOL domains, with significant reductions in physical function, mental health, and kidney disease-related QOL, highlighting the potential impact of peripheral artery disease on dialysis patients. These results were similar in both the bilateral low group and the unilateral ABI group.

### 3.4. Association of ABI ≤ 0.9 with Mortality

**Figure 3:**
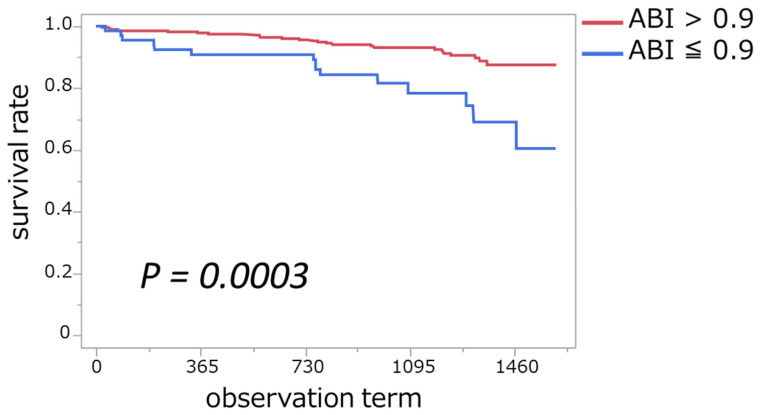
Cumulative mortality rate with an ABI ≤ 0.9 and with an ABI > 0.9. This figure shows the Kaplan–Meier survival curves comparing cumulative mortality rates between patients with an ABI ≤ 0.9 (blue line) and an ABI > 0.9 (red line). The survival rates are plotted over the observation term, with statistical significance assessed using the log-rank test (*p* = 0.0003).

During a median 3.3-year follow-up period, 40 patients died. The Kaplan–Meier method was utilized to compare survival times between dialysis patients with an ABI > 0.9 and those with an ABI ≤ 0.9, with the resulting survival curves depicted in Figure 3. The cumulative mortality rate was significantly higher in patients with an ABI ≤ 0.9 than in those with an ABI > 0.9 (6.6 vs. 2.5 per 100 patient-years, *p* < 0.001). Specifically, the mean survival time for patients with an ABI > 0.9 was 1275 days, whereas it was 1177 days for those with an ABI ≤ 0.9. Furthermore, Cox proportional hazards analysis demonstrated that patients with an ABI ≤ 0.9 had a significantly higher risk of mortality compared to those with an ABI > 0.9. The hazard ratio (HR) for mortality in the ABI ≤ 0.9 group was 3.09 (95% CI: 1.62–5.87, *p* < 0.001), indicating that the risk of death was approximately threefold higher in this group. This result remained statistically significant in the Wald test (*p* < 0.001), further supporting the prognostic value of the ABI in dialysis patients.

## 4. Discussion

In our analysis, we used the ABI as an assessment tool for arteriosclerosis to compare the QOL and found a significant relationship between the ABI and the QOL. Although a low ABI has been associated with a poorer prognosis in previous studies, our findings suggest that it is also linked to QOL impairment in hemodialysis patients [10]. There are reports indicating that improving the QOL leads to a better life prognosis in CKD patients [22], suggesting that the ABI could be a useful early detection tool for a declining QOL. This study presents several important strengths. Firstly, the utilization of the ABI as an early detection marker for QOL decline in dialysis patients is innovative, highlighting its potential clinical application. Secondly, the study includes a comprehensive evaluation of both physical and mental health aspects across multiple QOL domains, ensuring a holistic understanding of patient well-being. Thirdly, patients with a lower ABI had a lower phase angle and SMI, which may indicate potential sarcopenia and muscle attenuation. Lastly, the findings underscore the importance of the ABI as a predictor of survival, making it a valuable tool in the management of dialysis patients.

PAD affects over 200 million people worldwide, with CKD and dialysis being significant risk factors [23]. Patients undergoing maintenance hemodialysis progress to end-stage kidney disease (ESKD) following a period of CKD. As a result, these patients often present with risk factors such as hypertension, dyslipidemia, diabetes, and a history of smoking. These risk factors, presented even before the initiation of hemodialysis, contribute to vascular sclerosis and systemic endothelial damage due to chronic uremia, oxidative stress, and inflammatory conditions [24,25]. Endothelial dysfunction perpetuates a cycle of arteriosclerosis [26], impacting not only the kidneys but also other organs, thereby increasing the likelihood of complications such as coronary artery disease, cerebrovascular disease, and arteriosclerosis obliterans of the lower limbs. These atherosclerotic conditions further induce inflammation, which is a risk factor for CKD [27]. Moreover, advanced CKD itself is known to provoke inflammatory cytokines, leading to chronic systemic inflammation [28]. CKD patients frequently present these comorbidities and continue to experience chronic inflammation even after initiating dialysis. Chronic inflammation results in decreased skeletal muscle mass due to metabolic disorders, such as increased catabolism and uremia, progressing to a state of malnutrition, as exemplified by sarcopenia [29,30]. Our findings revealed a significant association between a decrease in the SMI and a phase angle and a lower ABI, based on body composition analysis. This suggests a correlation between arterial stiffness and nutritional disorders, including frailty and skeletal muscle mass. Although an ABI ≤ 0.9 is widely used as a diagnostic criterion for PAD, its applicability in dialysis patients requires careful consideration. In dialysis patients, arterial stiffness and vascular calcification may lead to falsely elevated ABI values, potentially underestimating the prevalence of PAD. However, previous studies have demonstrated that even in this population, an ABI ≤ 0.9 remains a useful marker for PAD risk assessment [31]. Given these findings, we adopted this threshold in our study while recognizing the need for further research to determine the optimal ABI cutoff for dialysis patients. Moreover, we found that patients with an ABI ≤ 0.9 had a lower prescription rate of phosphate binders and a higher frequency of vitamin D supplementation. Previous research has indicated that increased use of phosphate binders is associated with lower mortality rates and improved nutritional markers [32,33]. Phosphate binders have also been shown to play a role in reducing vascular calcification, LDL cholesterol, and CRP levels. Based on these findings, the reduced nutritional intake in patients with an ABI ≤ 0.9 may reduce the need for phosphate binders, potentially increasing the risk of vascular calcification and accelerating the progression of atherosclerosis. Although we did not measure blood vitamin D levels in this study, it is well-established that 87% of dialysis patients suffer from vitamin D deficiency. Vitamin D supplements are commonly used to treat hypocalcemia related to this deficiency [34]. Vitamin D deficiency has been associated with malnutrition, atherosclerosis, vascular calcification, and PAD [35]. The lower HbA1c levels in this group, despite a higher prevalence of diabetes, may be attributed to protein-energy wasting, a condition frequently observed in hemodialysis patients with PAD. PEW leads to muscle and fat loss, altered glucose metabolism, and increased insulin sensitivity due to muscle wasting, resulting in lower HbA1c levels [36]. Elevated ECW/ICW ratios, often observed in malnourished patients, correlate with higher NT-proBNP levels, indicating volume overload and heart failure [37,38]. In this study, similar findings were observed in patients with an ABI ≤ 0.9, suggesting that this imbalance in fluid distribution may reflect an underlying state of malnutrition. Body composition analysis and NT-proBNP measurements further support that patients with an ABI ≤ 0.9 are at greater risk for malnutrition and heart failure. This reduction in muscle mass, compounded by neuropathy caused by cerebrovascular disease related to arteriosclerosis, may lead to motor disorders, impaired swallowing function, and sensory disturbances. Progression to ASO in the lower limbs often accompanies pain and infection, making ambulation difficult and daily activities increasingly challenging, resulting in decreased ADL. This study suggests that ABI reduction in dialysis patients may be associated with a complex interplay of vascular, nutritional, and inflammatory factors, all of which collectively contribute to QOL deterioration. Therefore, the ABI should not only be considered as a marker of PAD but also as a broader indicator of systemic health impairment. In patients with PAD, particularly those who experience rest pain or have undergone lower limb amputations, phantom limb pain, a sense of loss, economic loss due to work limitations, and prolonged hospitalization all interrelate, likely causing holistic suffering [39]. Previous studies have investigated the direct relationship between ASO and QOL, but the ABI values in those studies were as low as 0.42, indicating more advanced vascular sclerosis compared to this study [40]. In the present study, the cutoff value for the ABI was set at 0.9, suggesting that even a less severe reduction in the ABI can significantly impact QOL. Our findings indicate that ABI reduction in dialysis patients may not reflect peripheral arterial occlusion but rather a multifactorial condition involving vascular calcification, arterial stiffness, hemodynamic instability, and malnutrition. These factors must be considered when interpreting ABI values in this population. The findings of this study suggest that the ABI could serve as an early indicator for declining QOL in dialysis patients. If an ABI reduction is observed, a multidisciplinary approach involving nephrologists, cardiologists, and nutritionists may be necessary to address potential contributors beyond arterial stenosis, such as nutritional deficiencies, systemic inflammation, and hemodynamic disturbances. In addition, collaborative efforts between patients and their caregivers to identify health problems in daily life can potentially enhance QOL [41].

This study has several limitations. First, as a single-country study (Japan), the findings may not be generalizable due to differences in dietary habits, healthcare systems, and lifestyle factors. Dietary variations, including protein and phosphate intake, may influence nutritional status and vascular health, potentially affecting ABI and QOL. Additionally, lifestyle factors such as physical activity levels and access to preventive healthcare could impact the association between the ABI and clinical outcomes. Future studies should explore these factors to improve generalizability. Second, despite adjusting for key clinical variables, residual confounding factors such as diabetes and other vascular diseases may have influenced the results. Factors such as malnutrition, inflammation, and fluid balance, which are common in dialysis patients, could contribute to both ABI reduction and QOL deterioration. Third, the cross-sectional and retrospective cohort design limits the ability to establish causal relationships, necessitating prospective studies with long-term follow-up. Fourth, the ABI was categorized using the ≤0.9 cutoff, but this threshold may not be optimal for dialysis patients [42]. Additionally, in cases of severe vascular calcification, ABI measurements may not accurately reflect arterial stenosis. As vascular imaging (e.g., ultrasound, CT angiography) was not performed, it remains unclear to what extent ABI reduction reflects true stenotic lesions versus other vascular dysfunctions such as endothelial dysfunction or arterial stiffness. Fifth, the ABI was measured in the non-shunt arm to minimize the impact of vascular access, but this differs from standard practices in non-dialysis populations, potentially affecting comparability. Sixth, QOL scores were self-reported, introducing potential response bias. Although trained staff explained the procedure to minimize bias, responses could be influenced by environmental factors (e.g., dialysis room vs. home) and cohabitants’ advice. Additionally, lower-limb symptoms, particularly in diabetic patients, may have affected the results [43]. To enhance generalizability, future studies should involve multi-center international collaborations to assess the ABI’s impact across diverse healthcare settings. Intervention strategies such as structured exercise programs, nutritional optimization, and intensive cardiovascular risk management should be evaluated in patients with a reduced ABI to determine whether targeted therapies can mitigate the observed decline in QOL and survival. Further research should explore the longitudinal effects of unilateral and bilateral ABI reduction on QOL and survival, incorporate vascular imaging for better assessment, and evaluate optimal ABI thresholds in dialysis patients through larger sample sizes and comparisons with non-dialysis populations.

## 5. Conclusions

This study highlights the significant association between a low ABI, reduced QOL, and a higher mortality risk in hemodialysis patients. Interestingly, the ABI was also linked to muscle weakness, nutritional disorders, and volume overload, suggesting its utility beyond PAD screening. Given its ease of measurement, routine ABI assessment could aid in early risk stratification and guide targeted interventions. Clinically, ABI measurement may help identify high-risk dialysis patients who would benefit from multidisciplinary interventions, such as individualized exercise programs, optimized nutritional support, and enhanced cardiovascular risk management. Integrating ABI assessment into routine dialysis care could facilitate earlier detection of functional decline, allowing for timely and tailored therapeutic strategies to improve both QOL and survival. Future research should refine ABI cutoff values for dialysis patients and evaluate its role in improving clinical outcomes.

## Figures and Tables

**Table 1:** Clinical parameters stratified by ABI ≤ 0.9 and >0.9.

Clinical Parameters	ABI ≤ 0.9 (n = 66, 19.1%)			ABI > 0.9 (n = 280, 80.9%)			*p*-Value
	Median/ Proportion	IQR	95% CI	Median/ Proportion	IQR	95% CI	
Age (years)	70	60–77	66–71	66	54–73	62–65	0.006
Male gender, %	76	n/a	64–84	70	n/a	65–75	0.38
DM, %	62.1	n/a	50–73	41.4	n/a	41–59	0.002
Cardiovascular disease, %	31.8	n/a	32–68	16.1	n/a	12–21	0.003
Smoking, %	25.8	n/a	17–37	26.1	n/a	21–32	0.96
HD duration (months)	100	61–226	116–172	61.5	30–117	79–98	<0.001
Body mass index, (kg/m^2^)	22.2	19.4–24.7	21.1–22.8	22.1	20.0–25.4	22.2–23.2	0.29
SBP (mmHg)	150	132–164	142–154	145	129–160	142–147	0.23
DBP (mmHg)	72	65–81	71–77	76	68–86	76–79	0.043
BUN (mg/dL)	53	44–62	51–58	58	49–70	59–62	0.002
Creatinine (mg/dL)	10.01	8.68–11.15	9.47–10.38	10.3	8.83–12.03	10.2–10.8	0.11
Calcium (mg/dL)	8.7	8.3–9.0	8.5–8.8	8.7	8.3–8.9	8.56–8.68	0.69
Phosphorus (mg/dL)	5.5	5.0–5.9	5.3–5.8	5.6	5.0–6.3	5.5–5.8	0.49
HbA1c (%)	5.9	5.4–6.7	5.8–6.4	6.3	5.7–6.8	6.3–6.6	0.041
iPTH (pg/mL)	160	98–208	140–186	157	106–217	158–180	0.68
CRP (mg/dL)	0.14	0.06–0.36	0.17–0.57	0.1	0.04–0.27	0.23–0.38	0.21
Hb (g/dL)	11.5	10.8–12.0	11.2–11.7	11.2	10.7–11.9	11.1–11.4	0.06
NT-proBNP (pg/mL)	6050	3550–17,750	8822–15,941	2985	1595–6700	5542–7981	<0.001
KT/Vurea (D)	1.84	1.68–2.09	1.83–1.99	1.80	1.63–2.04	1.82–1.9	0.19
GNRI	95	89–100	93–97	97	90–104	96–98	0.11
Number of any phosphate binders 0/1/2 or more	24/38/4	n/a	n/a	83/137/60	n/a	n/a	0.015
Calcitriol use, %	86.9	n/a	78–94	70.4	n/a	65–75	0.004

This table presents the comparison of various clinical parameters between patients with an ABI ≤ 0.9 and those with an ABI > 0.9. Median values or proportions, interquartile ranges (IQRs) and a 95% confidence interval (CI) are shown for each variable. “n/a” indicates that the IQRs could not be calculated due to the nature of the data. The Wilcoxon test or Pearson’s chi-square test were used to determine the statistical significance of differences, with corresponding *p*-values provided. Refer to the Abbreviations section for definitions of all abbreviations used in the table.

**Table 2:** Body fluid composition by ABI ≤ 0.9 and ABI > 0.9.

Body Fluid Composition	ABI ≤ 0.9 (n = 66, 19.1%)			ABI > 0.9 (n = 280, 80.9%)			*p*-Value
	Median	IQR	95% CI	Median	IQR	95% CI	
Total body water, L	32.6	27.5–35.4	30.4–33.2	33.3	28.0–38.9	32.8–34.5	0.10
Total body water, per BSA	19.6	18.3–20.6	19.0–19.9	20.0	18.5–21.7	19.8–20.3	0.038
Intracellular water, L	19.7	16.6–21.8	18.4–20.1	20.3	17.1–24.1	20.0–21.2	0.06
Intracellular water, L per BSA	11.8	11.0–12.6	11.5–12.0	12.2	11.3–13.3	12.1–12.4	0.006
Extracellular water, L	12.9	10.8–13.8	12.1–13.1	12.9	10.8–14.8	12.7–13.3	0.31
Extracellular water, L per BSA	7.7	7.3–8.1	7.5–7.9	7.8	7.2–8.4	7.7–7.9	0.49
Extracellular water-to-intracellular water ratio	0.66	0.63–0.67	0.65–0.66	0.63	0.61–0.63	0.63–0.64	<0.001
Phase angle, °	4.8	4.2–5.1	4.4–4.9	5.3	4.6–6.2	5.3–5.5	<0.001
Skeletal muscle mass index, kg/m^2^	8.4	7.7–9.3	6.5–7.1	9.0	8.0–10.1	7.0–7.4	0.007

This table presents the comparison of various body fluid composition parameters between patients with an ABI ≤ 0.9 and those with an ABI > 0.9. Median values, interquartile ranges (IQRs), and a 95% confidence interval (CI) are shown for each variable. The Wilcoxon test was used to determine the statistical significance of differences, with corresponding *p*-values provided. Refer to the Abbreviations section for definitions of all abbreviations used in the table.

**Table 3:** QOL domains by ABI ≤ 0.9 and ABI > 0.9.

QOL Domains	ABI ≤ 0.9 (n = 66, 19.1%)			ABI > 0.9 (n = 280, 80.9%)			*p*-Value
	Median	IQR	95% CI	Median	IQR	95% CI	
Physical functioning	65	45–75	52–65	80	65–95	72–78	<0.001
Role limitations caused by physical health problems	0	0–100	28–50	75	0–100	55–65	<0.001
Bodily pain	56	33–80	47–61	70	53–90	65–72	<0.001
General health	40	25–60	33–42	50	35–55	43–47	0.004
Vitality	45	25–60	36–48	55	40–75	53–59	<0.001
Social functioning	63	50–75	57–73	75	50–100	70–77	0.048
Role limitations caused by emotional health problems	0	0–100	32–55	100	0–100	58–69	0.002
Emotional well-being	68	48–81	53–67	68	56–84	64–69	0.17
Symptoms	79	64–86	60–75	83	73–92	78–82	0.003
Effects of kidney disease	78	53–88	59–74	78	66–91	72–77	0.10
Burden of kidney disease	31	13–45	27–40	38	25–50	36–41	0.039
Work status	25	0–50	21–38	50	0–100	44–54	<0.001
Cognitive function	87	73–100	67–84	93	80–100	85–90	0.017
Quality of social interaction	87	67–100	67–83	93	73–100	81–86	0.24
Sleep	58	47–73	48–60	63	48–75	57–62	0.25
Social support	67	67–83	58–73	67	67–83	68–74	0.51
Dialysis staff encouragement	75	50–88	61–76	75	50–100	57–62	0.99
Patient satisfaction	83	67–100	67–82	83	67–100	68–74	0.43

This table presents the comparison of QOL domain scores between patients with an ABI ≤ 0.9 and those with an ABI > 0.9. Median values and interquartile ranges (IQRs) are shown for each variable. The Wilcoxon test was used to determine the statistical significance of differences, with corresponding *p*-values provided. QOL domains were derived from the Kidney Disease Quality of Life (KDQOL) instrument, which comprehensively assesses physical, mental, and social well-being in patients undergoing dialysis. Refer to the Abbreviations section for definitions of all abbreviations used in the Table.

## Data Availability

The datasets generated and analyzed during the current study are available from the corresponding author on reasonable request.

## Abbreviations

The following abbreviations are used in this manuscript:

ABI	Ankle–brachial index
QOL	Quality of life
PAD	Peripheral arterial disease
DM	Diabetes mellitus
HD	Hemodialysis
SBP	Systolic Blood Pressure
DBP	Diastolic Blood Pressure
BUN	Blood urea nitrogen
HbA1c	Glycated Hemoglobin
iPTH	Intact parathyroid hormone
CRP	C-reactive protein
Hb	Hemoglobin
NT-proBNP	N-Terminal Pro B-Type Natriuretic Peptide
GNRI	Geriatric Nutritional Risk Index
ICW	Intracellular water
ECW	Extracellular water
SMI	Skeletal muscle mass index
BSA	Body surface area
